# Racial disparities among candidemic patients at a Southern California teaching hospital

**DOI:** 10.1017/ice.2023.74

**Published:** 2023-11

**Authors:** Victoria C. Grant, Anna Y. Zhou, Karen K. Tan, Jacinda C. Abdul-Mutakabbir

**Affiliations:** 1 Department of Pharmacy, Loma Linda University Medical Center, Loma Linda, California, USA; 2 Department of Pharmacy Practice, Loma Linda University School of Pharmacy, Loma Linda, California, USA; 3 Division of Clinical Pharmacy, Skaggs School of Pharmacy and Pharmaceutical Sciences, University of California San Diego, La Jolla, California, USA; 4 Divison of the Black Diaspora and African American Studies, University of California San Diego, La Jolla, California, USA

## Abstract

Racially and ethnically minoritized (REM) patients are disproportionately affected by infectious diseases, including candidemia. REM patients with candidemia were significantly younger, with trends toward more risk factors for candidemia and longer lengths of stay. Although *Candida parapsilosis* was more common in REM patients, there were no differences in mortality rates.

Racial and ethnic disparities within the US healthcare system have been well documented.^1^ Importantly, infectious diseases are a leading contributor to disproportionate rates of mortality observed among racially and ethnically minoritized (REM) patients.^2^ In the United States, ∼120,000 nosocomial bloodstream infections (BSIs) occur each year, and crude mortality rates range from 15% to 30%.^3^ Candidemia is a leading cause of nosocomial BSI, with all-cause inpatient mortality rates as high as 25%.^4^ The highest rates of mortality are observed among those infected with *Candida glabrata* and those with delayed initiation of antifungal therapy.^4–6^ The SENTRY Antifungal Surveillance Program database reports the *Candida albicans* remains the leading causative organism of candidemia; however, rates of infection secondary to non-*albicans* species, notably *C. parapsilosis*, are increasing.^5,7^

The Centers for Disease Control and Prevention surveillance data reports rates of candidemia are twice as high among Black patients compared to their White counterparts.^4^ Nonetheless, literature exploring differences among other REM patients with candidemia is limited. Determining whether differences exist among racial groups with candidemia and understanding the origins of such differences are critical to early diagnosis and initiation of optimal therapy in high-risk patients. Here, we describe infection characteristics and outcomes among REM and non–racially and ethnically minoritized (n-REM) patients with candidemia at our institution.

## Methods

### Study design, patient population, and location

In this retrospective, observational study, we evaluated adult patients with candidemia at 3 Loma Linda University hospitals from January 1, 2020, through December 31, 2021. Patients were included if they had ≥1 blood culture growing *Candida* species. Based on patient-reported race and ethnicity, patients were dichotomized into the REM or n-REM group. Patients identifying as White and non-Hispanic were included in the n-REM group. Although data for all other racial and ethnic groups (Hispanic/Latino, Black/African American, Asian, Alaskan Native, and unknown) were collected, these patients were aggregated into the REM group due to small sample sizes among the individual racial and ethnic groups. All hospitals are academic institutions located in San Bernardino County in Southern California, where the population is predominantly Hispanic and Latino. The study was approved by the institutional review board of Loma Linda University.

### Data collection and study definitions

Data regarding baseline demographics and comorbid conditions were collected. Risk factors for invasive candidiasis were identified and collected in accordance with established literature.^8^ Relevant laboratory data were collected on the day of blood-culture collection to determine severity of illness. Pertinent microbiological, treatment, and outcomes data were recorded.

Broad-spectrum antibiotic use was defined as *Pseudomonas* spp and/or methicillin-resistant *Staphylococcus aureus* coverage for ≥72 hours prior to blood-culture collection. The time to blood-culture positivity was defined as the time from blood-culture collection to the time yeast was identified by gram staining. An antifungal was considered to have in vitro activity if the minimum inhibitory concentration was interpreted as susceptible or susceptible-dose dependent according to Clinical and Laboratory Standards Institute (CLSI) M60 (June 2020) breakpoints. Time to initiation of in vitro active antifungal therapy was defined as the time from blood-culture collection to the time of initiation of in vitro active antifungal therapy. This metric was measured only for patients initiated on antifungal therapy following blood-culture collection.

### Statistical analysis

Study data were collected and managed using REDCap version 10.5.1 software (Vanderbilt University, Nashville, TN) electronic data capture tools hosted at Loma Linda University. Data analyses were conducted using SPSS version 28 software (IBM, Armonk, NY). Data were compared between the n-REM and REM groups using univariate analysis. Further analysis comparing individual racial and ethnic groups could not be performed due to limited sample sizes. Significance was defined as *P* ≤ .05.

## Results

### Study population

Select demographic, clinical, microbiological, treatment, and outcomes data are displayed in Table 1. Data for individual race and ethnicity groups are provided in the Supplementary Materials (online). Overall, 86 unique episodes of candidemia were included: 54 in the REM group and 32 in the n-REM group. In the REM group, 36 patients (66.7%) were Hispanic or Latino and 12 patients (22.2%) were non-Hispanic Black or African American. Racially and ethnically minoritized patients were significantly younger compared to their n-REM counterparts (mean age, 54 vs 62 years; *P* = .017). The median Charlson comorbidity index was similar between groups (median, 5 vs 5; *P* = .871).

**Table 1.** Select Demographic, Clinical, Microbiological, Treatment, and Outcomes Data Among REM and n-REM Patients with Candidemia

Characteristic	Total (N = 86), No. (%)^a^	n-REM (n = 32), No. (%)^a^	REM (n = 54), No. (%)^a^	*P* Value
Age, mean y (SD)	56.9 (17)	62.4 (13.4)	53.7 (18.1)	.017
Sex, male	47 (54.7)	19 (59.4)	28 (51.9)	.512
**Race and ethnicity**				N/A
Hispanic/Latino	36 (41.9)	…	36 (66.7)	
White non-Hispanic	32 (37.2)	32 (100)	…	
Black/African American non-Hispanic	12 (14)	…	12 (22.2)	
Alaskan Native	3 (3.5)	…	3 (5.6)	
Asian	2 (2.3)	…	2 (3.7)	
Other	1 (1.2)	…	1 (1.9)	
**Common risk factors (>25%)**				
Indwelling central line	63 (73.3)	22 (68.8)	41 (75.9)	.615
Diabetes mellitus	44 (51.2)	19 (59.4)	25 (46.3)	.271
Broad-spectrum antibiotic use	39 (45.3)	12 (37.5)	27 (50)	.274
ICU admission	39 (45.3)	12 (37.5)	27 (50)	.274
Total parenteral nutrition	27 (31.4)	9 (28.1)	8 (33.3)	.641
**Select baseline demographics**				
Liver disease	25 (29.1)	13 (40.6)	12 (22.2)	.088
Chronic kidney disease	22 (25.6)	8 (25)	14 (25.9)	1
Hematologic malignancy	3 (3.5)	1 (3.1)	2 (3.7)	1
Nonhematologic malignancy	10 (11.6)	2 (6.3)	8 (14.8)	.31
Neutropenia	2 (2.3)	…	2 (3.7)	.527
Solid organ transplant	8 (9.3)	1 (3.1)	7 (13)	.249
Active immunosuppressive/immune-modulator use	7 (.1)	2 (6.3)	5 (9.3)	1
Sepsis or septic shock	73 (84.9)	26 (81.3)	47 (87)	.343
ICU at time of BC collection	48 (55.8)	17 (53.1)	31 (57.4)	.823
APACHE II score, median (IQR)	(n = 41)	(n = 16)	(n = 25)	.341
	29 (21.5–36.5)	30.5 (26–43.7)	28 (21–33.5)	
SOFA score, median (IQR)	(n = 40)	(n = 160	(n = 24)	.747
	11.5 (7–14.7)	12.5 (7–15.7)	10 (7–14)	
Quick SOFA score, median (IQR)	(n = 38)	(n = 15)	(n = 23)	.426
	1 (0.75–2)	2 (0–3)	1 (1–2)	
Infectious diseases consult	79 (91.9)	28 (87.5)	51 (94.4)	.416
**Suspected source**				.236
Central line	43 (50)	14 (43.8)	29 (53.7)	
Intra-abdominal	14 (16.3)	5 (15.6)	9 (16.7)	
Translocation	6 (7)	1 (3.1)	5 (9.3)	
Genitourinary	4 (4.7)	4 (12.5)	…	
Other	7 (8.1)	2 (6.2)	5 (9.3)	
Unknown	12 (14)	6 (18.8)	6 (11.1)	
Attempted source control	49 (57)	18 (56.3)	31 (57.4)	.765
Time to BC positivity, median h (IQR)	39.5 (32–59)	43 (32–67.7)	38.5 (29.7–58)	.823
Time to in vitro active antifungal, median h (IQR)^b^	(n = 77)	(n = 26)	(n = 51)	.399
	48 (35–64.5)	44 (35.7–69)	48 (34–62)	
Micafungin DOT, median d (IQR)	5 (2–13)	5 (3–12)	6 (2–13)	.704
Antifungal DOT, median d (IQR)	10 (5.5–17)	11 (5–15.5)	9.5 (6–18)	.934
Ophthalmologic consultation or exam	65 (75.6)	21 (65.6)	44 (81.5)	.122
All-cause, inpatient mortality	42 (48.8)	16 (50)	26 (48.1)	.414
Hospital length of stay, median f (IQR)	22 (12–34.2)	19.5 (7.2–18.5)	25.5 (14–36)	.093
ICU length of stay, median d (IQR)	(n = 64)	(n = 24)	(n = 40)	.439
	15.5 (7–28)	14 (4–22)	16.5 (8–32)	

Note. APACHE, Acute Physiology and Chronic Health Evaluation; BC, blood culture; DOT, duration of therapy; ICU, intensive care unit; IQR, interquartile range; SD, standard deviation; SOFA, Sequential Organ Failure Assessment.

a Units unless otherwise specified.

b Time to initiation of in vitro active antifungal therapy was only determined for patients initiated on empiric antifungal therapy following BC collection.

Patients in the REM group presented with more risk factors for candidemia, although this difference did not reach statistical significance (4 vs 3 risk factors per patient; *P* = .13). Importantly, REM patients demonstrated trends toward higher rates of indwelling central lines at the time of blood-culture collection (75.9% vs 68.8%; *P* = .615) and receipt of total parenteral nutrition prior to diagnosis (33.3% vs 28.1%; *P* = .641).

We did not detect statistically significant differences in severity of illness. Most patients presented in sepsis or septic shock (87% vs 81.3%; *P* = .343), and more than half of all included patients were admitted to an ICU at the time of blood-culture collection (57.4% vs 53.1%; *P* = .823). Overall, central-line infection was the most common source of candidemia. A trend toward increased rates of central-line infection was observed among REM patients (53.7% vs 43.8%; *P* = .236).

### Microbiology and treatment

**Fig. 1.**
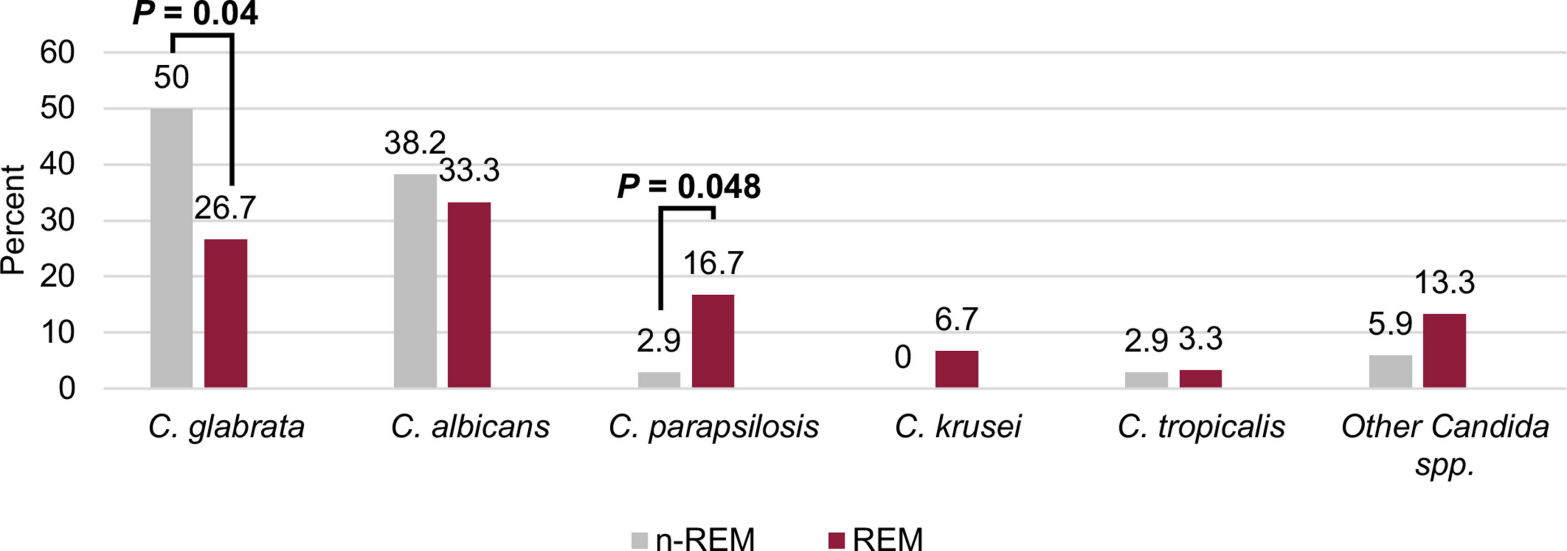
Microbiological Distribution

In this cohort, 7 patients (8.1%) had polymicrobial candidemia. In total, 94 *Candida* isolates were identified across the entire cohort. Despite overall similar documented sources of infection, significant microbiological differences were observed (Fig. 1). *Candida glabrata* was more often isolated in n-REM patients (27% vs 50%; *P* = .04), whereas *C. parapsilosis* was more often isolated in REM patients (17% vs 3%; *P* = .048). Of the 11 *C. parapsilosis* isolates identified among the entire cohort, 3 (27%) were resistant to fluconazole according to CLSI breakpoints. The median time to blood culture positivity was shorter in the REM group, but this difference was not statistically significant (38.5 vs 43 hours; *P* = .823).

The median time to initiation of in vitro active antifungal therapy was numerically longer among REM patients (48 vs 44 hours; *P* = .399). Rates of attempted source control and infectious diseases consultation were similar between groups.

## Outcomes

The median hospital length of stay (LOS) was numerically longer among REM patients (25.5 vs 19.5 days; *P* = .093). The median ICU LOS was slightly longer among REM patients, but this difference did not reach statistical significance (16.5 vs 14 days; *P* = .439). Overall, all-cause inpatient mortality was 48.5%, and rates were similar between groups (48.1% vs 50%; *P* = .414).

## Discussion

Candidemia is associated with a high mortality burden and has been shown to disproportionately affect minoritized patients.^4^ Notably, our study population of REM patients was largely comprised of Hispanic and Latino patients, subsequently representing a population for which data of this nature are extremely limited. We noted significant differences in microbiological distribution among REM and n-REM patients. *Candida parapsilosis* was more frequently isolated from REM patients and *C. glabrata* was more frequently isolated from n-REM patients. This finding is concerning because rates of azole-resistant *C. parapsilosis* are globally increasing.^7^ In our study, 27% of *C. parapsilosis* isolates were resistant to fluconazole per CLSI breakpoints. Notably, when compared to other species, *C. parapsilosis* is more commonly associated with the development of biofilms on foreign material, higher affinity for parenteral nutrition, and lower mortality rates.^5,10^ The increased isolation of *C. parapsilosis* from REM patients in our study may be explained by high rates of central-line infection and receipt of TPN observed in the REM group. Nevertheless, the possibility of increased risk of candidemia due to *C. parapsilosis* among REM patients would be concerning given increasing rates of azole resistance. Fortunately, overall rates of all-cause mortality in our study were similar between groups, despite the significantly higher infection rates with *C. parapsilosis* among REM patients.

Although no differences were observed in all-cause inpatient mortality, REM patients were significantly younger and demonstrated a trend toward longer total and ICU LOS. Secondary to systemic racism, REM patients are disproportionately affected by structural and social determinants of health, ultimately resulting in increased incidence and severity of acute and chronic conditions.^1,9^ Unfortunately, our data support previous studied that have demonstrated increased severity of illness among infected REM patients.^9^

This study had several limitations. First, the overall limited sample size may limit the interpretability of our results and the ability draw strong conclusions on the causality of the observed outcomes. Limited sample sizes among individual racial and ethnic groups inhibited our ability to perform robust analyses and required the development of a single heterogenous comparator group (REM). However, our findings can serve as a basis for hypothesis generation and designing larger prospective studies in the future. Secondly, the retrospective nature of this study limited our ability to validate self-reported race and ethnicity, to identify other social vulnerabilities in our patient population, or to determine causality regarding differences in baseline demographics and risk factors.

Further research is needed to investigate the potential differences in infection characteristics and outcomes between REM and n-REM patients, utilizing larger sample sizes and a more comprehensive approach that considers the impact of social determinants of health and systemic, institutional, and/or interpersonal racism on outcomes. Furthermore, emphasis should also be placed on utilizing results to conduct quality improvement projects and implement relevant antimicrobial stewardship measures.

## Supplementary material

For supplementary material accompanying this paper visit https://doi.org/10.1017/ice.2023.74. click here to view supplementary material
