# Supplementary material for: Racial disparities among candidemic patients at a Southern California teaching hospital
Source: Infect Control Hosp Epidemiol. 2023 Apr 24;44(11):1866–9. doi: 10.1017/ice.2023.74 (PMC10665870; doi:10.1017/ice.2023.74)
Supplement: Supplementary file 1 [file S0899823X23000740sup001.docx]

**Supplemental Table 1. Select Demographic, Clinical, Microbiological, Treatment, and Outcomes Data Among Patients with Candidemia: Disaggregated by Race/Ethnicity**

|  | Total (*n* = 86) | n-REM (*n* = 32) | REM (*n* = 54) | | | | |  |
| --- | --- | --- | --- | --- | --- | --- | --- | --- |
|  |  |  | Hispanic/ Latino (*n* = 36) | Black/AA  (*n* = 12) | Asian  (*n* = 2) | Alaskan native  (*n* = 3) | Unknown  (*n* = 1) |  |
| Age (years), median (IQR) | 58 (44-69) | 59.50 (52-73.25) | 60 (38.5-68.7) | 46 (32.5-69) | 66.50 | 37 | 56 |  |
| Male gender, *n* (%) | 47 (54.7) | 19 (59.4) | 23 (63.9) | 4 (33.3) | - | - | 1 (100) |  |
| Comorbidities/Risk Factors, *n* (%) | | | | | | | | |
| Indwelling central line | 63 (73.3) | 22 (68.8) | 26 (72) | 10 (83.3) | 1 (50) | 3 (100) | 1 (100) |  |
| Diabetes mellitus | 44 (51.2) | 19 (59.4) | 18 (50) | 5 (41.7) | 1 (50) | - | 1 (100) |  |
| Broad spectrum antibiotic use | 39 (45.3) | 12 (37.5) | 21 (58.3) | 5 (41.7) | - | - | 1 (100) |  |
| ICU admission | 39 (45.3) | 12 (37.5) | 23 (63.9) | 2 (16.7) | 1 (50) | 1 (33.3) | - |  |
| Total parenteral nutrition | 27 (31.4) | 9 (28.1) | 9 (25) | 5 (41.7) | 2 (100) | 3 (100) | 1 (100) |  |
| Liver disease | 25 (29.1) | 13 (40.6) | 12 (33.3) | - | - | - | - |  |
| Chronic kidney disease | 22 (25.6) | 8 (25) | 11 (30.6) | 3 (25) | - | - | - |  |
| Hematologic malignancy | 3 (3.5) | 1 (3.1) | 1 (2.8) | 1 (8.3) | - | - | - |  |
| Non-hematologic malignancy | 10 (11.6) | 2 (6.3) | 4 (11.1) | 1 (8.33) | 1 (50) | - | - |  |
| Neutropenia | 2 (2.3) | - | 2 (5.6) | - | - | - | - |  |
| Solid organ transplant | 8 (9.3) | 1 (3.1) | 5 (13.9) | 2 (16.7) | - | - | - |  |
| Active immunosuppressive/ immune-modulator use | 7 (.1) | 2 (6.3) | 4 (11.1) | 1 (8.3) | - | - | - |  |
| Sepsis/septic shock, *n* (%) | 73 (84.9) | 26 (81.3) | 33 (91.7) | 8 (66.7) | 2 (100) | 3 (100) | 1 (100) |  |
| ICU at time of BC collection, *n* (%) | 48 (55.8) | 17 (53.1) | 24 (66.7) | 5 (41.7) | 1 (50) | - | 1 (100) |  |
| APACHE II score, median (IQR) | *n* = 41  29 (21.5-36.5) | *n* = 16  30.5 (26-43.7) | *n* = 20  27.5 (21-33.7) | *n* = 3  29 (n/a) | *n* = 1  34 (n/a) | *n* = 0  - | *n* = 1  17 (n/a) |  |
| SOFA score, median (IQR) | *n* = 40  11.5 (7-4.7) | *n* = 16  12.5 (7-15.7) | *n* = 19  9 (7-14) | *n* = 3  13 (n/a) | *n* = 1  13 (n/a) | *n* = 0  - | *n* =1  5 (n/a) |  |
| Quick SOFA score, median (IQR) | *n* = 38  1 (0.75-2) | *n* = 15  2 (0-3) | *n* = 12  1 (0-2) | *n* = 7  1 (1-1) | *n* = 1  3 (n/a) | *n* = 3  2 (n/a) | *n* = 0  - |  |
| Infectious diseases consult, *n* (%) | 79 (91.9) | 28 (87.5) | 34 (94.4) | 12 (100) | 1 (50) | 3 (100) | 1 (100) |  |
| Suspected Source, *n* (%) | | | | | | | |  |
| Central line | 43 (50) | 14 (43.8) | 19 (52.8) | 7 (58.3) | 1 (50) | 2 (66.7) | - |  |
| Intra-abdominal | 14 (16.3) | 5 (15.6) | 6 (16.7) | 1 (8.3) | - | 1 (33.3) | 1 (100) |  |
| Translocation | 6 (7) | 1 (3.1) | 3 (8.3) | 2 (16.7) | - | - | - |  |
| Genitourinary | 4 (4.7) | 4 (12.5) | - | - | - | - | - |  |
| Other | 7 (8.1) | 2 (6.2) | 3 (8.3) | 2 (16.7) | - | - | - |  |
| Unknown | 12 (14) | 6 (18.8) | 5 (13.9%) | - | 1 (50) | - | - |  |
| Attempted source control, *n* (%) | 49 (57) | 18 (56.3) | 19/28 (67.9) | 8/9 (88.9) | - | 3 (100) | 1 (100) |  |
| Time to BC positivity (hours), median (IQR) | 39.5 (32-59) | 43 (32-67.7) | 46.5 (29.2-59) | 37 (31-40) | 64 (n/a) | 28 (n/a) | 34 (n/a) |  |
| Time to *in vitro* active antifungal (hours), median (IQR)* | *n* = 77  48 (35-64.5) | *n* = 26  44 (35.7-69) | *n* = 34  51 (35.5-66.7) | *n* = 12  44.5 (36-61.5) | *n* = 1  93 (n/a) | *n* = 3  26 (n/a) | *n* = 1  0 (n/a) |  |
| Micafungin DOT (days), median (IQR) | 5 (2-13) | 5 (3-12) | 5 (2-9) | 11 (1.2-14.7) | 2 (n/a) | 6 (n/a) | 50 (n/a) |  |
| Antifungal DOT (days), median (IQR) | 10 (5.5-17) | 11 (5-15.5) | 9 (5-20) | 13.5 (7-15) | 2 (n/a) | 10 (n/a) | 50 (n/a) |  |
| Ophthalmologic consult/exam, *n* (%) | 65 (75.6) | 21 (65.6) | 28 (77.8) | 11 (91.7) | 1 (50) | 3 (100) | 1 (100) |  |
| All-cause, inpatient mortality *n* (%) | 42 (48.8) | 16 (50) | 22 (61.1) | 3 (25) | 1 (50) | - | - |  |
| Hospital length of stay (days), median (IQR) | 22 (12-34.2) | 19.5 (7.2-28.5) | 25 (14-34.7) | 25.5 (16.5-38.7) | 19.5 (n/a) | 10 (n/a) | 73 (n/a) |  |
| ICU length of stay (days), median (IQR) | *n* = 64  15.5 (7-28) | *n* = 24  14 (4-22) | *n* = 31  17 (8-32) | *n* = 6  13 (3.7-23) | *n* = 1  34 (n/a) | *n* = 1  15 (n/a) | *n* = 1  18 (n/a) |  |

AA: African American; APACHE: Acute Physiology and Chronic Health Evaluation; BC: blood culture; DOT: duration of therapy; ICU: intensive care unit; IQR: interquartile range; SD: standard deviation; SOFA: Sequential Organ Failure Assessment

*Time to initiation of in vitro active antifungal therapy was only determined for patients initiated on empiric antifungal therapy following BC collection.
